# Occult papillary thyroid carcinoma with cystic nodal metastasis mimicking a thyroglossal duct cyst: A case report

**DOI:** 10.1016/j.ijscr.2019.11.028

**Published:** 2019-11-27

**Authors:** Alexander Mimery, Mohamed Al-Askari

**Affiliations:** Gladstone Hospital, Park Street, Gladstone, QLD, 4680, Australia^1^

**Keywords:** Case report, General surgery, Endocrine surgery, Papillary thyroid carcinoma, Thyroglossal duct cyst

## Abstract

**Introduction:**

The approach to the anterior neck lump involves the consideration of numerous benign and malignant differentials.

**Presentation of case:**

We present a rare case of an occult papillary thyroid cancer with cystic nodal metastases presenting as a thyroglossal duct cyst.

**Discussion:**

We explore the literature surrounding occult papillary thyroid carcinoma/microcarcinomas and the potential for the disease to present as a cervical lump. There is discussion surrounding the diagnostic and therapeutic difficulties associated with this case, and the importance of lateral thinking and clinical flexibility.

**Conclusion:**

We recommend that the management approach to any anterior midline neck lump involve a comprehensive work-up including radiological imaging, routine histopathological analysis, and the early involvement of the subspecialty multidisciplinary team.

## Highlights

• Occult thyroid cancer may present as nodal metastases mimicking benign disease.
• A comprehensive workup of any neck lump is essential to exclude sinister pathology.
• Early MDT involvement is vital when there is a lack of widely accepted guidelines.

## 1 Introduction

Papillary thyroid carcinoma (PTC) is the most common form of thyroid cancer disproportionally affecting females in their fourth to sixth decade of life [1]. There is a tendency for PTC to develop cystic changes, or have cystic lymph node metastasis [2]. Cystic lymph node metastasis may pose a diagnostic challenge given their similarity with benign alternatives, thus delaying or preventing appropriate treatment.

Thyroglossal duct cysts (TDC) are a common cause for a midline neck mass and typically arise due to a persistence of the thyroglossal duct during early development [3]. Issues associated with this pathology includes poor cosmesis, recurrent infections, fistula formation [4]. Additionally, there is a small (<1 %) chance of developing a thyroglossal duct carcinoma [5]. Papillary thyroid carcinoma is the most common malignancy (92 %) found in TDC, with less common variants being squamous cell carcinoma (5.2 %) and follicular carcinoma (1.7 %) [6].

Other differentials for the midline neck mass includes, epidermoid cysts, or ectopic thyroid/parathyroid tissue. Diagnostic ambiguity may result in difficulties in implementing an optimal treatment strategy.

The case report described here is in line with the SCARE criteria [7].

## 2 Presentation of case

A 34-year old female was referred to a regional surgical clinic after noticing a small anterior midline neck mass over the last two weeks. She did not have any other significant past medical or surgical history.

**Fig. 1:**
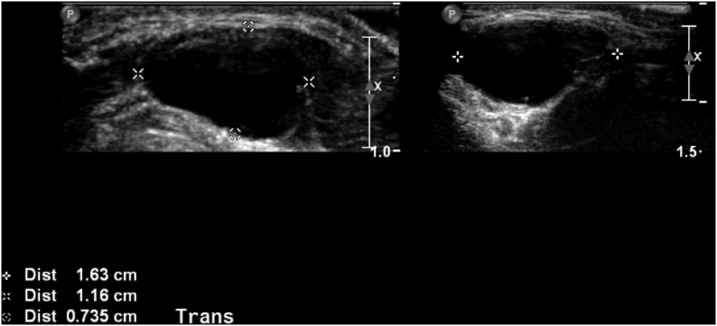
An ultrasound scan of the anterior midline neck mass. A well-defined 16 × 12 × 7 mm avascular thin walled cystic structure is demonstrated.

An ultrasound (US) of the neck demonstrated a well-defined 16 × 12 × 7 mm avascular thin walled cystic lesion with few internal debris (Fig. 1). The thyroid was sonographically unremarkable. On examination, the mass measured 2 × 2 cm and moves during swallowing or protrusion of the tongue. The thyroid examines normally, and there is no palpable cervical lymphadenopathy. The clinical and radiological findings at this stage were consistent with a thyroglossal cyst.

A sistrunk procedure was performed without any complications. Intraoperatively, the appearance and location of the cystic structure was consistent with a TDC given its close proximity adjacent to the mid hyoid bone. The patient recovered well post operatively and was discharged the next day.

**Fig. 2:**
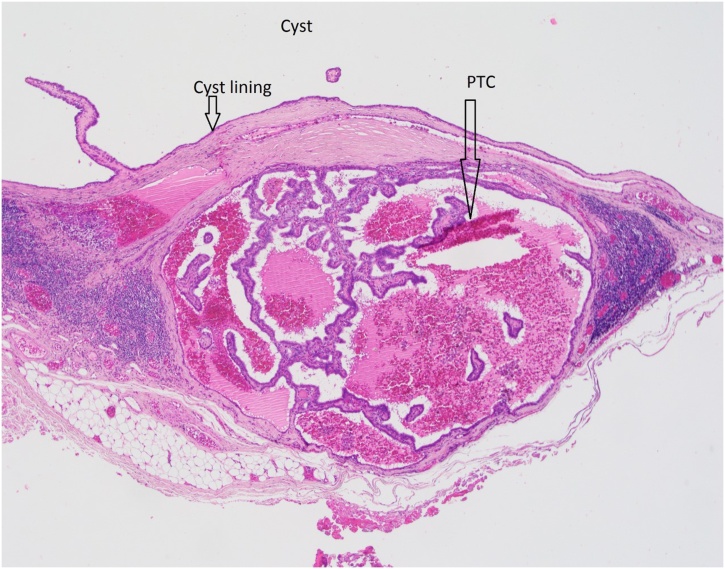
An annotated hematoxylin and eosin histological slide demonstrating a cystic structure containing papillary thyroid carcinoma (PTC).

Histological review of the resected specimen demonstrated nodal tissue with metastatic papillary thyroid carcinoma (Fig. 2). A cystic component was also identified - however it is unclear whether this originated from a TDC, or is due to a cystic lymph node metastasis.

The patient was subsequently referred to a tertiary endocrine multidisciplinary meeting for consideration of further management options.

**Fig. 3:**
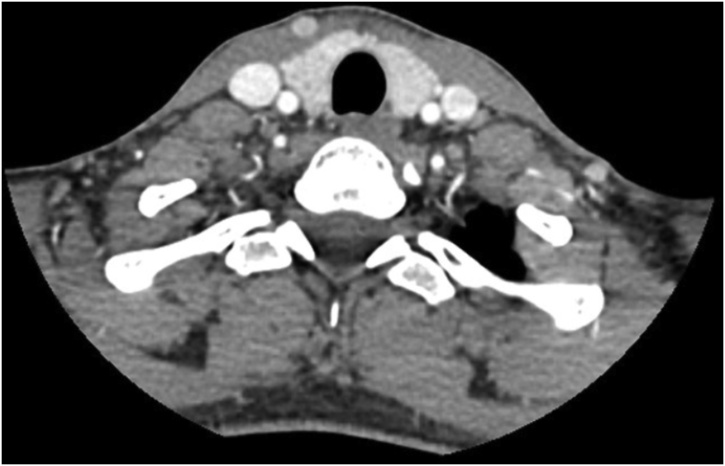
A contrast CT axial scan of the thyroid gland demonstrating no obvious pathology.

Staging investigations were organised to exclude a primary thyroid gland malignancy and other regional metastases. CT neck demonstrated an unremarkable thyroid gland (Fig. 3). It was noted that two small nodes within the left jugular chain demonstrated some minor peripheral enhancement, however they were not enlarged. Repeat US thyroid demonstrated identified a small 5 × 5 mm nodule within the upper pole of the left thyroid gland. The remainder of gland appeared unremarkably.

An US guided fine needle aspirate biopsy of the jugular chain lymph node was performed. The histology demonstrated a benign reactive lymph node with no evidence of malignancy.

The final consensus was that the resected specimen most likely represented a cystic nodal metastasis of a PTC. Further surgery was recommended to exclude an occult primary thyroid gland neoplasm.

A total thyroidectomy and level two, three, and six neck dissection was performed without any intraoperative or acute postoperative concerns. The histology of the thyroid gland demonstrated four papillary microcarcinomas ranging between 0.45 mm and 4.5 mm. The largest lesion (4.5 mm) anatomically correlated with the left upper lobe lesion visualised on the repeat ultrasound. All lesions were intrathyroidal with clear surgical margins, and without any high-risk histology. A total of 31 lymph nodes were removed. All nodes were negative for malignancy. Two parathyroid glands were reimplanted into the sternocleidomastoid.

Adjuvant therapy was not required given the favourable histology. The patient recovered well post operatively and was commenced on thyroid hormone supplementation.

## 3 Discussion

The management approach to a neck lump will vary significantly depending on the suspected pathology, and warrants a comprehensive workup to narrow down the extensive list of differentials. Our case highlights the diagnostic difficulties associated with this presentation, and emphasizes the importance of lateral thinking and clinical flexibility.

The term ‘occult PTC’ may refer to several distinct clinical scenarios, but is often considered synonymous with papillary thyroid microcarcinoma (PTMC) [8]. PMTC is a subset of PTC and is defined as disease measuring less than or equal to 1 cm in diameter [9]. The prevalence of PTMC within autopsy series are 100–1000 times higher than clinically detected disease [10], suggesting that the majority of microcarcinomas remain latent. Nevertheless, there are cases within literature where occult PTC presents as a neck lump secondary to nodal metastases [11]. These cases are often mistaken for benign disease more commonly found within the region, thus resulting in a suboptimal treatment approach. To our knowledge, this is only the second described case of an occult PTC with cystic nodal metastasis mimicking a thyroglossal duct cyst [12].

Although the incidence of malignancy found within thyroglossal duct cysts is <1 % [5], we recommend the routine histopathological review of all resected specimens. Thyroglossal duct cysts are typically lined with either respiratory (columnar to stratified cuboidal) epithelium (38 %), squamous epithelium (10 %), or a combination of both types (51 %). A sistrunk procedure is the preferred surgical approach for uncomplicated TDC due to its lower risk of recurrence [13]. If a malignancy is identified then staging investigations are indicated to exclude metastatic disease. Additional therapies may be undertaken depending on the extent of the malignancy – including a thyroidectomy, neck dissection, or radioactive iodine. However, there are no widely accepted guidelines available and management is largely driven by clinician acumen.

We encountered a degree of diagnostic ambiguity differentiating between the cystic metastasis of an occult PTC with a thyroglossal duct carcinoma (TGDC), however it is noted that the histological presence of nodal tissue is classically not associated with a TGDC.

We recommend the early involvement of a specialist thyroid multidisciplinary team when approaching a rare, clinically ambiguous scenario. It was eventually recommended that an aggressive surgical approach be adopted, given the patients young age and excellent premorbid condition. Such a strategy proved invaluable given the finding of several microcarcinomas within the native thyroid gland.

## 4 Conclusion

Ultimately, this is a rare case of an occult PTC with cystic nodal metastases mimicking a benign thyroglossal duct cyst. We recommend that the management approach to any anterior midline neck lump involve a comprehensive work-up including radiological imaging, routine histopathological analysis, and the early involvement of the subspecialty multidisciplinary team.

## Sources of funding

This research did not receive any specific grant from funding agencies in the public, commercial, or not-for-profit sectors.

## Ethical approval

This report was granted exemption from Human Research Ethics Committee review by the chair of the Central Queensland Hospital and Health Service Human Research Ethics Committee and (reference number HREC/16/QCQ/31).

## Informed consent

Written informed consent was obtained from the patient for the development and publication of this case report.

## Author’s contribution

Dr Alexander Mimery is the primary author of this case report. Dr Mohamed Al-Askari was the patient’s surgeon, and was supervised the writing of this case report.

## Registration of research studies

This is not applicable due to the nature of this case study.

## Guarantor

Dr Alexander Mimery (the primary author) is the guarantor of this work.

## Provenance and peer review

Not commissioned, externally peer-reviewed.

## Declaration of Competing Interest

The authors have nothing to declare.
